# Detection and Tracking of a Moving Target Using SAR Images with the Particle Filter-Based Track-Before-Detect Algorithm

**DOI:** 10.3390/s140610829

**Published:** 2014-06-19

**Authors:** Han Gao, Jingwen Li

**Affiliations:** School of Electronic and Information Engineering, Beihang University, Beijing 100191, China; E-Mail: lijingwen@buaa.edu.cn

**Keywords:** particle filter (PF), track-before-detect (TBD), detection, tracking, synthetic aperture radar (SAR)

## Abstract

A novel approach to detecting and tracking a moving target using synthetic aperture radar (SAR) images is proposed in this paper. Achieved with the particle filter (PF) based track-before-detect (TBD) algorithm, the approach is capable of detecting and tracking the low signal-to-noise ratio (SNR) moving target with SAR systems, which the traditional track-after-detect (TAD) approach is inadequate for. By incorporating the signal model of the SAR moving target into the algorithm, the ambiguity in target azimuth position and radial velocity is resolved while tracking, which leads directly to the true estimation. With the sub-area substituted for the whole area to calculate the likelihood ratio and a pertinent choice of the number of particles, the computational efficiency is improved with little loss in the detection and tracking performance. The feasibility of the approach is validated and the performance is evaluated with Monte Carlo trials. It is demonstrated that the proposed approach is capable to detect and track a moving target with SNR as low as 7 dB, and outperforms the traditional TAD approach when the SNR is below 14 dB.

## 1. Introduction

Detection and tracking of a moving target with synthetic aperture radar (SAR) systems is a challenging problem, which has drawn an increasing attention recently. Its achievement requires the combination of a specially designed measurement mode for acquiring more than a single frame of observations, with a suitable signal processing method for estimating the trajectory based on all observations. Current approaches to detecting and tracking a moving target with SAR systems are mainly achieved with the track-after-detect (TAD) scheme, based on the acquisition of multi-frame SAR images [1–4]. The concept of this scheme is to detect the target at a plot level at first, then associate the detections and estimate the trajectory by passing through a tracker filter. It is effective when the target has a high signal-to-noise ratio (SNR) [5]. However, when the SNR is relative low, such as those of spaceborne SAR images, it is difficult for the target to cross a standard detection threshold, which makes the detection and tracking fail [6]. In comparison, the track-before-detect (TBD) scheme, using the entire output as measurement for the tracker and avoiding the thresholding process of detection, allows for the detection and tracking of low SNR targets with good performance [7]. Therefore, there is a great incentive to exploit TBD algorithms to detect and track moving targets with SAR system, when the SNR of the target is low so that TAD algorithms fail. Multiple TBD algorithms have been developed previously, including batch methods, such as the Hough transform, dynamic programming (DP) and maximum likelihood techniques, and recursive methods based on the Bayesian approach, such as the particle filter (PF) [8]. Among all of the algorithms, the DP-based TBD algorithm and PF-based algorithm are widely used for solving problems of radar target detection and tracking under low SNR conditions. As a batch method, the DP-based TBD algorithms, while effective, generally require discretization of the state space and are very computationally intensive. To solve the problem, some modified algorithms were proposed recently to improve computational efficiency [9,10]. The PF-based TBD algorithm was introduced to the radar system in [8,11]. To make it more efficient and more accurately match radar signal processing, improvements have been made on the PF-based TBD algorithm by Rutten [7,12]. More recent investigations of the algorithm have focused on its application in different radar systems, such as over-the-horizon radar (OTHR) [13], passive radars [14] and asynchronous multiple radars [15]. Due to the non-linear and non-Gaussian characteristics of the SAR image measurement with regard to the target state, the PF-based TBD algorithm proposed by Rutten is applied for detection and tracking in SAR system in this paper.

An approach to detection and tracking of moving targets using SAR images with the PF-based TBD algorithm is proposed and evaluated in this paper, with improvements on the computational efficiency, which are all validated by the simulations via Monte Carlo trials. The SAR measurement model is incorporated into the PF-based TBD algorithm for the first time, which is thus capable of resolving the ambiguity in target azimuth position and radial velocity while tracking. The feasibility of the approach is demonstrated on simulated multi-frame SAR images and the performance is evaluated. Compared with the traditional TAD approach, the advantages in the detection and tracking of low SNR targets is illustrated. Improvements on the computational efficiency are achieved with the usage of the sub-area for calculation of the likelihood ratio and a pertinent choice of the number of particles, which are also validated by simulations with little loss in the detection and tracking performance.

To focus on the process of detecting and tracking a low SNR target, two assumptions have been made in this paper. Firstly, it is assumed that multi-frame SAR images are available, which show the same scene at different times. Even though the moving target detection is achieved by multi-looking at multi-channel SAR [16,17], it is hard to track the moving target without a sequence of time-series images from a long-time and continuous observation. Since a novel persistent staring mode SAR is proposed by Small Business Innovation Research (SBIR) to enable the detection and long-duration tracking of targets [18], multi-frame SAR images in this paper are expected to be a sequence of single-look SAR images generated from a single-channel persistent staring mode SAR. Among these images, the clutter background appears static, whereas the positions of the moving target change from image to image. Secondly, it is assumed that the clutter among multi-frame SAR images has been completely suppressed, which means the only interference considered here is the noise. The clutter suppression is expected to be achieved with change detection (CD) method, which has been fully studied and extensively used already [19].

This paper is organized as follows: in Section 2, the target dynamic model and the system measurement model are set up, and the likelihood ratio is calculated. The approach to detection and tracking of the moving target with the PF-based TBD algorithm is presented in Section 3. The performance of the approach is analyzed in Section 4, and improvements on the efficiency are also presented. Simulation results are presented in Section 5. Finally, Section 6 gives the conclusions.

## 2. Models

### 2.1. Target Dynamic Model

Considering a point target moving in the X-Y plane, the target state at time instant *k*, is denoted as $x_{k}={\left[\begin{array}{cccc} x_{k} & {\dot{x}}_{k} & y_{k} & {\dot{y}}_{k} \end{array}\right]}^{T}$, where (*x_k_*, *y_k_*) and (*ẋ_k_*, *ẏ_k_*) are respectively the position and velocity of the target. A constant-velocity movement model is adopted in this paper, thus the evolution of the state is modeled by a linear stochastic process:

(1) $$x_{k+1}=Fx_{k}+w_{k},E_{k}=e$$

where **F** is the state transition matrix given by:

(2) $$F=\left[\begin{array}{cccc} 1 & T & 0 & 0\\ 0 & 1 & 0 & 0\\ 0 & 0 & 1 & T\\ 0 & 0 & 0 & 1 \end{array}\right]$$

and *T* is the frame period. **w***_k_* is the Gaussian distributed process noise with zero mean and covariance matrix **Q** given by:

(3) $$Q=q\left[\begin{array}{cccc} T^{3}/3 & T^{2}/2 & 0 & 0\\ T^{2}/2 & T & 0 & 0\\ 0 & 0 & T^{3}/3 & T^{2}/2\\ 0 & 0 & T^{2}/2 & T \end{array}\right]$$

where *q* is the process noise coefficient [20]. The random variable *E_k_*∈{*e,ē*} indicates the existence or non-existence of the target and evolves according to a two state Markov chain, which is defined as:

(4) $$\prod =\left[\begin{array}{cc} 1-P_{b} & P_{b}\\ P_{d} & 1-P_{d} \end{array}\right]$$

Here, *P_b_* = Prob{*E_k_*=*e*|*E_k_*_–1_=*ē*} is the probability of target birth and *P_b_* = Prob{*E_k_*=*ē*|*E_k_*_–1_=*e*} is the probability of target death.

### 2.2. System Measurement Model

To apply the TBD scheme to the SAR system, the intensity maps of multi-frame SAR images are used as measurements. The measurement **z***_k_* at each time instant *k*, consists of the set of intensities in each bin of the *k*th frame image with *N_a_* bins in azimuth and *N_r_* bins in range, which is:

(5) $$z_{k}=\left\{\left|z_{k}^{\left(i,j\right)}\right|:i=1,\ldots ,N_{a},j=1,\ldots ,N_{r}\right\}$$

The measurement model in the cell (*i*, *j*) under target present or absent hypothesizes can, in general, be defined as follows:

(6) $$z_{k}^{\left(i,j\right)}=\{\begin{array}{ll} \left|h_{k}^{\left(i,j\right)}(x_{k})+v_{k}^{\left(i,j\right)}\right| & ,E_{k}=e\\ \left|v_{k}^{\left(i,j\right)}\right| & ,E_{k}=\bar{e} \end{array}$$

where *h*(·) is the measurement function of the moving target for the SAR system. $v_{k}^{\left(i,j\right)}$is the measurement noise in the cell (*i*, *j*), which is assumed to be independent from cell to cell and from frame to frame and zero mean Gaussian distributed with the variance σ^2^.

According to [21], there are three types of signal model determined by the velocity of target. For targets with small velocities [21], $h_{k}^{\left(i,j\right)}(x_{k})$ can be expressed as:

(7) $$h_{k}^{\left(i,j\right)}(x_{k})=\sigma \mathrm{sinc}\left\{\pi B_{a}\left[\frac{x_{i}}{V}-\frac{\left(x_{k}-\frac{{\hat{\dot{y}}}_{k}}{V}{\hat{y}}_{k}\right)}{V}\right]\right\}\mathrm{sinc}\left\{\pi B_{r}\left[\frac{2r_{j}}{c}-\frac{2\sqrt{{y_{k}}^{2}+h^{2}}}{c}\right]\right\}$$

where *x_i_* and *r_j_* correspond to the azimuth and range bins of the image, *B_a_* and *B_r_* are the Doppler bandwidth and the transmitted liner frequency modeled (LFM) signal bandwidth, respectively, *h* is the platform height, *V* denotes the velocity of SAR platform, and *c* is the velocity of light. For targets with large velocities, there are other two functions available [21], which can be exploited according to the velocity of the target of interest and are not discussed here.

### 2.3. Likelihood Ratio Calculation

Under the models set up above, it is reasonable to assume that the intensity of each bin is Ricean distributed, if there is a target present in noise, or Rayleigh distributed, if there is noise only [22]. Thus, in a given bin (*i*, *j*), the signal and noise likelihood functions are:

(8) $$\begin{array}{l} \begin{array}{c} p(z_{k}^{\left(i,j\right)}|x_{k},e_{k})=\frac{2z_{k}^{\left(i,j\right)}}{\sigma^{2}}\exp\left\{-\frac{{\left[z_{k}^{\left(i,j\right)}\right]}^{2}+{\left[h^{\left(i,j\right)}(x_{k})\right]}^{2}}{\sigma^{2}}\right\}I_{0}\left(\frac{2z_{k}^{\left(i,j\right)}h^{\left(i,j\right)}(x_{k})}{\sigma^{2}}\right) \end{array}\\ p(z_{k}^{\left(i,j\right)}|{\bar{e}}_{k})=\frac{2z_{k}^{\left(i,j\right)}}{\sigma^{2}}\exp\left\{-\frac{{\left[z_{k}^{\left(i,j\right)}\right]}^{2}}{\sigma^{2}}\right\} \end{array}$$

Since the noise in each bin is assumed to be independent, the complete likelihood function of the measurement is a product over all of the contributions:

(9) $$\begin{array}{l} p(z_{k}|x_{k},e_{k})=\overset{N_{a}}{\underset{i=1}{\text{∏}}}\overset{N_{r}}{\underset{j=1}{\text{∏}}}p(z_{k}^{\left(i,j\right)}|x_{k},e_{k})\\ p(z_{k}|{\bar{e}}_{k})=\overset{N_{a}}{\underset{i=1}{\text{∏}}}\overset{N_{r}}{\underset{j=1}{\text{∏}}}p(z_{k}^{\left(i,j\right)}|{\bar{e}}_{k}) \end{array}$$

Therefore, the corresponding statistical likelihood ratio can be calculated as [12]:

(10) $$L(z_{k}|x_{k},e_{k})=\frac{p(z_{k}|x_{k},e_{k})}{p(z_{k}|{\bar{e}}_{k})}=\prod_{i=1}^{N_{a}}\prod_{j=1}^{N_{r}}\exp\left\{-\frac{{\left[h^{\left(i,j\right)}(x_{k})\right]}^{2}}{\sigma^{2}}\right\}I_{0}\left(\frac{2z_{k}^{\left(i,j\right)}h^{\left(i,j\right)}(x_{k})}{\sigma^{2}}\right)$$

## 3. Method

In this section, a description of the PF-based TBD algorithm is given here, with further details referred to in [12]. The derivation of the TBD filter is given first, and the procedure to implement PF is provided.

### 3.1. TBD Filter Derivation

The TBD filter is to determine the target existence and estimate the target state, based on all observations via Bayesian recursion. Since the posterior density of target state is necessary to estimate the target state, it is crucial to derive the posterior target state density *p*(**x***_k_*|*e_k_*,**z**_1:k_) and the probability of existence *p*(*e_k_*|**z**_1:k_) from the TBD filter.

Expanded over the existence of the target at the previous frame, *p*(**x***_k_*|*e_k_*,**z**_1:k_) can be written as the weighted sum of two densities, the first one describing the continuing density, and the other describing the birth density:

(11) $$p(x_{k}|e_{k},z_{1:k})=p(x_{k}|e_{k},e_{k-1},z_{1:k})p(e_{k-1}|e_{k},z_{1:k})+p(x_{k}|e_{k},{\bar{e}}_{k-1},z_{1:k})p({\bar{e}}_{k-1}|e_{k},z_{1:k})$$

The continuing density can be written in terms of the likelihood ratio defined in Equation (10):

(12) $$p(x_{k}|e_{k},e_{k-1},z_{1:k})=\frac{L(z_{k}|x_{k},e_{k})p(x_{k}|e_{k},e_{k-1},z_{1:k-1})}{L(z_{k}|e_{k},e_{k-1},z_{1:k-1})}$$

The predicted density, *p*(**x***_k_*|*e_k_*,*e_k_*_–1_**,z**_1:k–1_), in the numerator of Equation (12) can be written in terms of the target dynamic model:

(13) $$p(x_{k}|e_{k},e_{k-1},z_{1:k-1})=\int p(x_{k}|x_{k-1},e_{k},e_{k-1})p(x_{k-1}|e_{k-1},z_{1:k-1})dx_{k-1}$$

and the likelihood ratio, *L*(**z***_k_*|*e_k_*,*e_k_*_–1_**,z**_1:k–1_), in the denominator of Equation (12) can be seen as a normalizing term, which results in:

(14) $$p(x_{k}|e_{k},e_{k-1},z_{1:k})\propto L(z_{k}|x_{k},e_{k})\int p(x_{k}|x_{k-1},e_{k},e_{k-1})p(x_{k-1}|e_{k-1},z_{1:k-1})dx_{k-1}$$

On the other hand, the birth density is given similarly by:

(15) $$p(x_{k}|e_{k},{\bar{e}}_{k-1},z_{1:k})\propto L(z_{k}|x_{k},e_{k})p(x_{k}|e_{k},{\bar{e}}_{k-1})$$

where *p*(**x***_k_*|*e_k_*,*ē_k_*_−1_) is the prior density describing a target which started to exist from Frame *k*.

Meanwhile, the mixing terms in Equation (11) can be factorized using Bayes' rule in the same way:

(16) $$\begin{array}{l} p(e_{k-1}|e_{k},z_{1:k})\propto L(z_{k}|e_{k},e_{k-1},z_{1:k-1})p(e_{k}|e_{k-1})p(e_{k-1}|z_{1:k-1})\\ p({\bar{e}}_{k-1}|e_{k},z_{1:k})\propto L(z_{k}|e_{k},{\bar{e}}_{k-1})p(e_{k}|{\bar{e}}_{k-1})p({\bar{e}}_{k-1}|z_{1:k-1}) \end{array}$$

where the likelihood ratio in this case can be written as:

(17) $$\begin{array}{l} L(z_{k}|e_{k},e_{k-1},z_{1:k-1})=\int L(z_{k}|x_{k},e_{k})p(x_{k}|e_{k},e_{k-1},z_{1:k-1})dx_{k}\\ L(z_{k}|e_{k},{\bar{e}}_{k-1})=\int L(z_{k}|x_{k},e_{k})p(x_{k}|e_{k},{\bar{e}}_{k-1})dx_{k} \end{array}$$

*p*(*e_k_*|**z**_1:_*_k_*) is also expanded over the existence of target at the previous frame in the same fashion to Equation (11), which results in:

(18) $$\begin{array}{ll} p(e_{k}|z_{1:k}) & =p(e_{k},e_{k-1}|z_{1:k})+p(e_{k},{\bar{e}}_{k-1}|z_{1:k})\\ & \propto L(z_{k}|e_{k},e_{k-1},z_{1:k-1})p(e_{k}|e_{k-1})p(e_{k-1}|z_{1:k-1})\\ & +L(z_{k}|e_{k},{\bar{e}}_{k-1})p(e_{k}|{\bar{e}}_{k-1})p({\bar{e}}_{k-1}|z_{1:k-1}) \end{array}$$

Summarizing these formulations above, the desired terms can be calculated as functions of:

◆ the prior probability of existence at Frame *k* − 1, *p*(*e_k_*_−1_|**z**_1:_*_k_*_−1_);
◆ the prior posterior target state density at Frame *k* − 1, *p*(**x***_k_*_−1_|*e_k_*_−1_,**z**_1:_*_k_*_−1_);
◆ the Markov transition terms, *p*(*e_k_*| *e_k_*_−1_) and *p*(*e_k_*|*ē_k_*_−1_);
◆ a transition density assuming that the target continued to exist through Frame *k* − 1 and *k*, *p*(**x***_k_*|**x***_k_*_−1_, *e_k_*,*e_k_*_−1_);
◆ a prior state density assuming that the target started to exist between Frame *k* − 1 and *k*, *p*(**x***_k_*|*e_k_*,*ē_k_*_−1_);
◆ the likelihood ratio *L*(**z***_k_*|**x***_k_*,*e_k_*).

All of the above are quantities that have been defined by the models set up in the previous section, or acquired during the recursion.

### 3.2. PF Implementation

The PF implementation of the above derivation is to represent the density by a set of random particles with associated weights, update particle locations and corresponding weights recursively with new observed measurement and eventually estimate based on these particles and weights [23,24]. To alleviate degeneracy, a resampling step is always performed. According to [25], the algorithmic description of the PF is provided here briefly. It incorporates four dependent processes to estimate the target state and the probability of existence, with a mixture of two sets of particles. Here, the probability of existence is calculated as a separate process for a better performance [26]:

(1) Create a set of *N_b_* birth particles sampled from the proposal density, by placing the particles randomly in several highest intensity cells of the data [26]:
  (19) $$x_{k}^{(b)i}\sim q(x_{k}|e_{k},{\bar{e}}_{k-1},z_{k}),\{i=1,\ldots ,N_{b}\}$$
  Then calculate the weights of these birth particles based on the likelihood ratio in Equation (10) and the proposal density:
  (20) $${\tilde{\omega }}_{k}^{(b)i}=L(z_{k}|x_{k}^{(b)i},e_{k})\frac{p(x_{k}^{(b)i}|e_{k},{\bar{e}}_{k-1})}{N_{b}q(x_{k}^{(b)i}|e_{k},{\bar{e}}_{k-1},z_{k})}$$
(2) Create a set of *N_c_* continuing particles using the target dynamic model in Equation (1) as the proposal function:
  (21) $$x_{k}^{(c)i}\sim q(x_{k}|x_{k-1},e_{k},e_{k-1},z_{k}),\{i=1...N_{c}\}$$
  Then calculate the weights as follows:
  (22) $${\tilde{\omega }}_{k}^{(c)i}=\frac{1}{N_{c}}L(z_{k}|x_{k}^{(c)i},e_{k})$$
(3) Calculate the mixing probabilities using sums of the weights:
  (23) $$\begin{array}{l} {\tilde{M}}_{c}=p(e_{k}|e_{k-1})p(e_{k-1}|z_{1:k-1})\sum_{i=1}^{N_{c}}{\tilde{\omega }}_{k}^{(c)i}=(1-P_{d})\cdot p(e_{k-1}|z_{1:k-1})\sum_{i=1}^{N_{c}}{\tilde{\omega }}_{k}^{(c)i}\\ {\tilde{M}}_{b}=p(e_{k}|{\bar{e}}_{k-1})p({\bar{e}}_{k-1}|z_{1:k-1})\sum_{i=1}^{N_{b}}{\tilde{\omega }}_{k}^{(b)i}=P_{b}\cdot p({\bar{e}}_{k-1}|z_{1:k-1})\sum_{i=1}^{N_{b}}{\tilde{\omega }}_{k}^{(b)i}z \end{array}$$
  Then calculate the probability of existence at Frame *k*, in terms of mixing probabilities:
  (24) $$p(e_{k}|z_{1:k})=\frac{{\tilde{M}}_{c}+{\tilde{M}}_{b}}{{\tilde{M}}_{c}+{\tilde{M}}_{b}+P_{d}p(e_{k-1}|z_{1:k-1})+(1-P_{b})p({\bar{e}}_{k-1}|z_{1:k-1})}$$
(4) Scale the particle weights according to the mixing probabilities:
  (25) $$\begin{array}{l} \omega_{k}^{(c)i}=\frac{\left(1-P_{d})p(e_{k-1}|z_{1:k-1}\right)}{{\tilde{M}}_{c}+{\tilde{M}}_{b}}{\tilde{\omega }}_{k}^{(c)i}\\ \omega_{k}^{(b)i}=\frac{P_{b}p({\bar{e}}_{k-1}|z_{1:k-1})}{{\tilde{M}}_{c}+{\tilde{M}}_{b}}{\tilde{\omega }}_{k}^{(b)i} \end{array}$$
  Then combine the two sets of particles into one large set:
  (26) $$\left\{\left(x_{k}^{(j)i},\omega_{k}^{(j)i})|i\in \{1,\ldots ,N_{j}\},j\in \{c,b\right\}\right\}$$
  and resample the large set of particles from *N_b_*+*N_c_* down to *N_c_*:
  (27) $$\left\{\left({\hat{x}}_{k}^{i},{\hat{\omega }}_{k}^{i})|i\in \{1,\ldots ,N_{c}\right\}\right\}$$
  After the above steps, *p*(**x***_k_*|*e_k_*,**z**_1_**_:_***_k_*) is approximated by the particles with uniform weights.
  Given a threshold *P_th_*, the target is declared present if the *p*(e*_k_*|**z**_1:_*_k_*) is over *P_th_*. The estimation of target state at Frame *k* is calculated via:
  (28) $${\tilde{x}}_{k}=\sum_{i=1}^{N_{c}}{\hat{\omega }}_{k}^{i}{\hat{x}}_{k}^{i}$$

## 4. Performance Analysis

The per-frame detection sensitivity, the estimation accuracy and the computation requirement [27–29] are used as the measures of performance (MOPs) in this paper. The per-frame detection sensitivity is the detected-frame rate as a function of SNR for a prescribed false-report rate. The estimation accuracy is the root mean square (RMS) position estimation error as a function of SNR for a prescribed false-report rate. Unlike that analyzed in the single-frame SAR image [30] for the purpose of detection and estimation, the estimation error here is averaged over detected frames in the context of tracking. The computation requirement is the central processing unit (CPU) time resource required to execute the algorithm and is measured as the CPU time in seconds elapsed during the execution of one Monte Carlo trial. All measures are averaged over multiple Monte Carlo trials. It should be noticed that the simulations are carried out on a computer with Xeon E5649 2.53 GHz processors and 12 GB RAM, and all programs were coded and run in MATLAB.

SNR directly related to the target amplitude is the dominant factor that determines the first two measures. Theoretically, as the SNR increases, the detection sensitivity and the estimation accuracy improve. It is validated by the simulation results in Section 5.2.

Since critics of the PF mainly hang on its computational intensiveness, improvements in the efficiency are necessary for the PF implementation. Due to the inherent characteristic of the SAR measurement function, the sub-area is substituted for the whole area to calculate the likelihood ratio as the most time-consuming step in the PF process, calculation of weights, depends mainly on the calculation of the likelihood ratio [31]. Theoretically, the likelihood ratio is calculated on all cells, as seen in Equation (10). However, the unique *h*(·) provides an opportunity to exclude most of the cells for calculation, since its major power only takes up minor cells surrounding the peak. In the image space, a rectangular region is substituted for the whole area for the efficient calculation of the likelihood ratio. Therefore, the likelihood ratio can be approximated by:

(29) $$\begin{array}{ll} L(z_{k}|x_{k},e_{k}) & \approx \underset{(i,j)\in S}{\text{∏}}\exp\left\{-\frac{{\left[h^{\left(i,j\right)}(x_{k})\right]}^{2}}{\sigma^{2}}\right\}I_{0}\left(\frac{2z_{k}^{\left(i,j\right)}h^{\left(i,j\right)}(x_{k})}{\sigma^{2}}\right)\\ & =\overset{N_{wa}}{\underset{i=1}{\text{∏}}}\overset{N_{wr}}{\underset{j=1}{\text{∏}}}\exp\left\{-\frac{{\left[h^{\left(i,j\right)}(x_{k})\right]}^{2}}{\sigma^{2}}\right\}I_{0}\left(\frac{2z_{k}^{\left(i,j\right)}h^{\left(i,j\right)}(x_{k})}{\sigma^{2}}\right) \end{array}$$

where *S* is the sub-area with size *N_wa_* × *N_wr_* used for the efficient calculation of the likelihood ratio. It is determined by the power of *h*(·) in the sub area, *P_sub_*, which is normalized by the power in the whole area. However, the efficient calculation method also results in degradation in the detection and tracking performance.

A pertinent choice of the number of particles is also crucial in the efficient application of the PF. Theoretically, the more number of particles is sampled, the more CPU time is required. However, a large amount of particles is necessary to achieve a good detection and an accurate estimation.

As analyzed above, computational efficiency benefits from a small size of the sub-area and a small number of particles, which are both validated by the simulation results in Section 5.3. However, these improvements in the efficiency are made by sacrificing detection and tracking performance. Therefore, it is significant to achieve a tradeoff between accuracy and efficiency in practice.

## 5. Simulation Results

The simulations are based on 20 ideal frames of clutter-free SAR images, with system parameters of the spaceborne SAR in [21]. A single point target appears at Frame 4 and disappears at Frame 17 with constant velocity (4 m/s, 4 m/s). For the sake of efficiency, images with 200 × 32 bins extracted from the entire images with 2,048 × 2,048 bins are used, *q* = 0.01, and *P_b_* = *P_d_* = 0.1. 12,500 particles are used for the birth and continuing densities each, for a total of 25,000 particles. All simulation results are averaged over 100 Monte Carlo trials.

### 5.1. Feasibility of the Method

**Figure 1.**
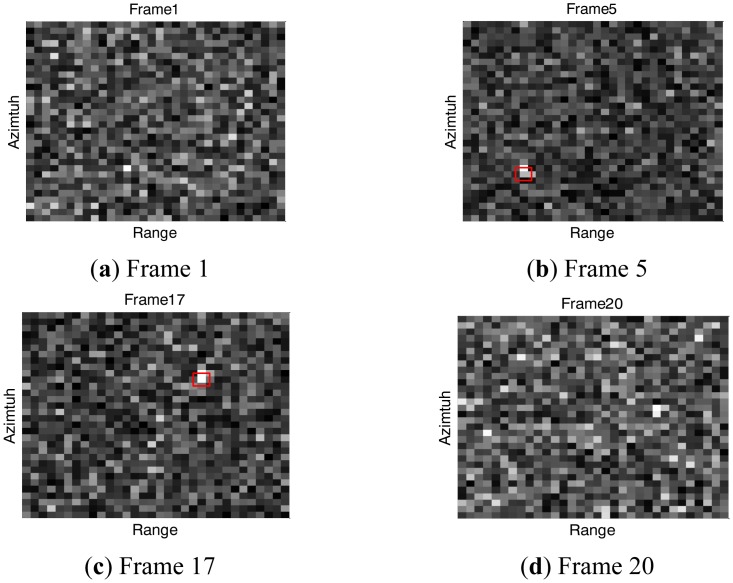
(**a**) Sampled image at Frame 1; (**b**) Sampled image at Frame 5; (**c**) Sampled image at Frame 17; (**d**) Sampled image at Frame 20.

**Figure 2.**
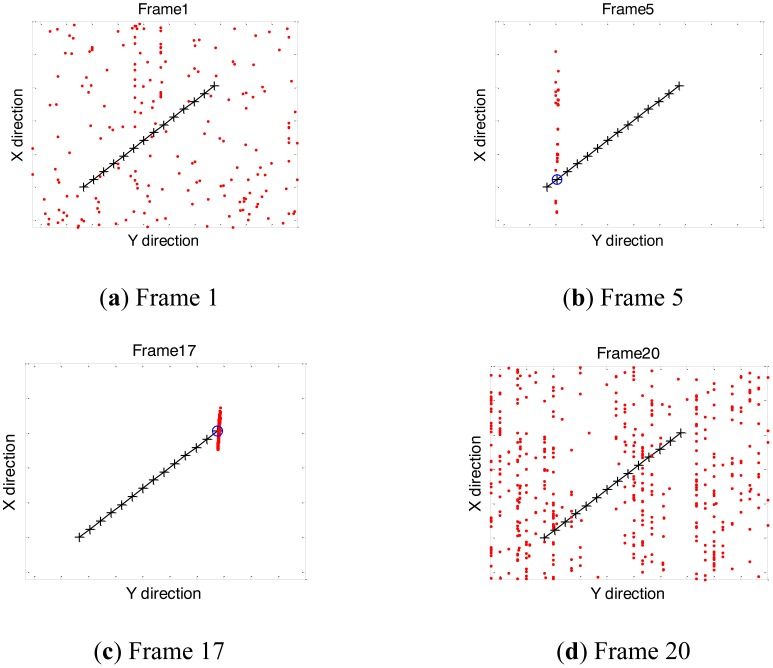
(**a**) Locations of particle clouds after filtering at Frame 1; (**b**) Locations of particle clouds after filtering at Frame 5; (**c**) Locations of particle clouds after filtering at Frame 17; (**d**) Locations of particle clouds after filtering at Frame 20.

Four sampled images with SNR set to 10 dB, are shown in Figure 1 in different frames: Frame 1, Frame 5, Frame 17 and Frame 20. The target exists at Frame 5 and Frame 17 at the red marked locations, but is indistinguishable from the background noise. The filtering results of the corresponding frames, are shown through the particle clouds in Figure 2, where the red points denote particle clouds, the blue circle marks the target position in the current frame if the target is present, and the black pluses compose the trajectory in the image space from Frame 4 to Frame 17.

Initially, there is no target at Frame 1 so that particles are randomly distributed over the observed area, which can be seen from Figure 2a. At Frame 5, particles condense to a slanted line in the azimuth direction but not the target location, as seen from the Figure 2b. The phenomenon indicates that the target has been tracked but the ambiguity in target azimuth position and radial velocity has not been resolved. It is shown in Figure 2c that particles condense to the target location at Frame 17, with the ambiguity resolved and the true estimation obtained. At Frame 20, as the target disappears, it is seen from Figure 2d the particles are randomly distributed over the area again.

**Figure 3.**
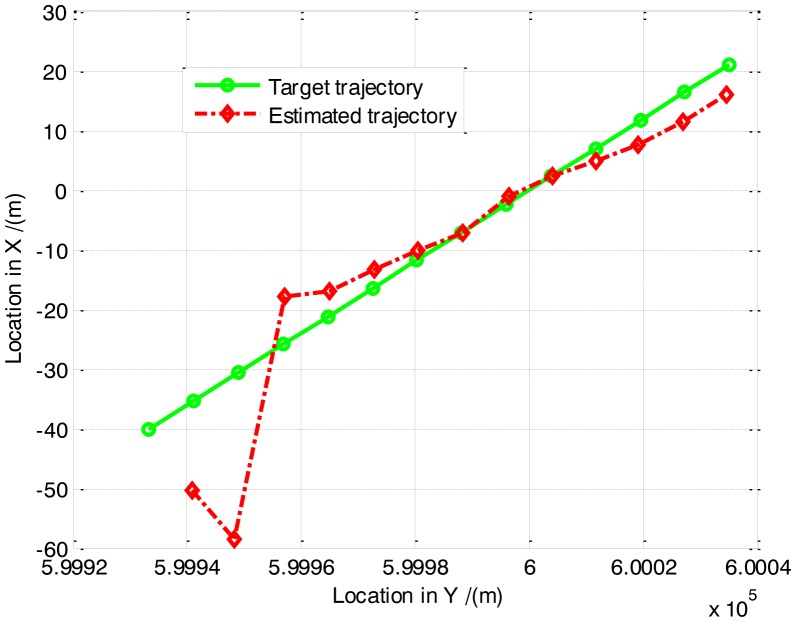
Tracking result of the PF-based TBD algorithm.

The true and estimated trajectories in 2-D space are shown in Figure 3. With the increase of frames of SAR images, the estimated trajectory converges to the true one gradually. It is demonstrated that the proposed approach is viable to detect and track the moving target, and capable to resolve the ambiguity in target azimuth position and radial velocity.

### 5.2. Performance Evaluation

The performance of the PF-based TBD algorithm is evaluated in simulations with the MOPs defined above. For the purpose of comparison, the performance of the traditional KF-based TAD algorithm, to detect the moving target with the constant false alarm rate (CFAR) algorithm and track with the KF [1,32], is also evaluated. Set *P_th_* = 0.5 [24] for the TBD algorithm, and the false alarm rate equal to 10^−6^ for the TAD algorithm.

**Figure 4.**
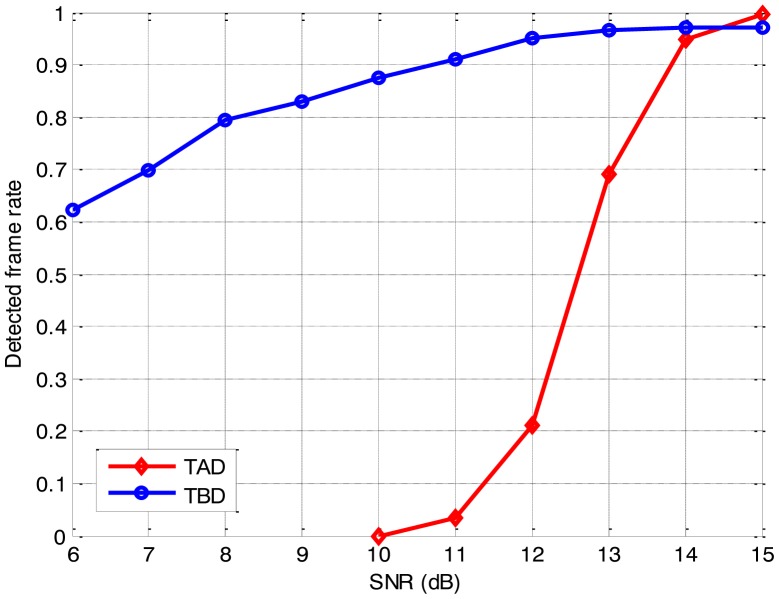
Detection sensitivity for the different algorithms.

**Figure 5.**
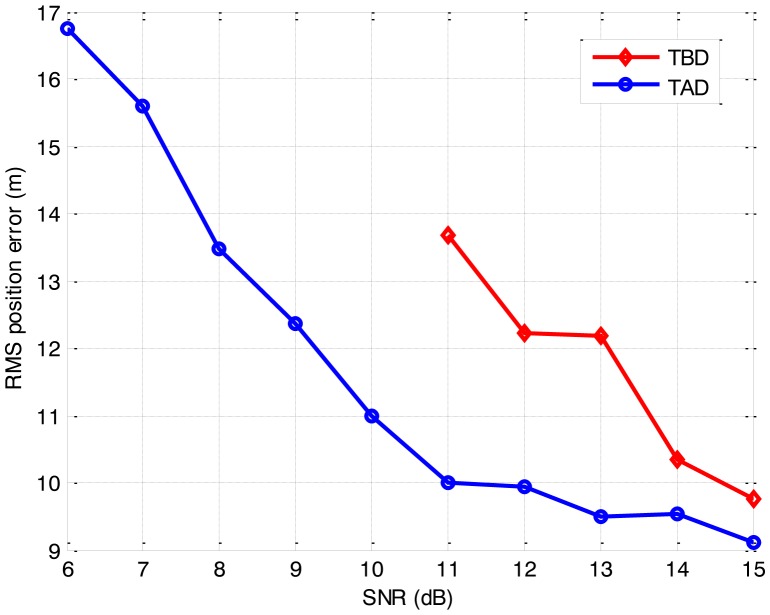
Estimation accuracy of the different algorithms.

The false-report rates and the computation requirements of the two algorithms are compared in Table 1. The detection sensitivity and the estimation accuracy with corresponding false-report rates are shown in Figures 4 and 5, respectively.

As anticipated, the detection sensitivity and the estimation accuracy of the proposed approach degrade as the SNR reduces. It is demonstrated that the TBD algorithm is capable of achieving a detected-frame rate of 0.7 for the false-report rate of 0.005, and maintains an acceptable RMS position error, even though the SNR is as low as 7 dB. Meanwhile, the TAD algorithm suffers a poor detection performance and an irresponsible RMS position error, when the SNR is below 13 dB. Compared to the TAD algorithm, the TBD algorithm shows a much larger computation requirement, but also a larger detectable and trackable SNR range and a better performance on the low SNR target. It is seen that the TBD algorithm outperforms the TAD algorithm on detection when the SNR is below 14 dB, and the estimation accuracy of the TBD algorithm is always superior to that of the TAD algorithm. In practical use, the problem of the large computational requirement can be solved by the optimization techniques [33,34] and parallel architectures [35,36], as the graphics processing unit (GPU) and general processing GPU (GPGPU) hardware have already been used for the efficient implementation of the PF [33,36] and achieve significant speedup ratio. Therefore, it is concluded that the proposed approach is capable to detect and track the moving target with SNR low as 7 dB, and it outperforms the traditional TAD algorithm when the SNR is below 14 dB.

### 5.3. Improvement on the Efficiency

Effects of the size of sub area and the number of particles on the performance, respectively, are illustrated in this part, with choices based on the tradeoff between efficiency and accuracy presented as well.

**Figure 6.**
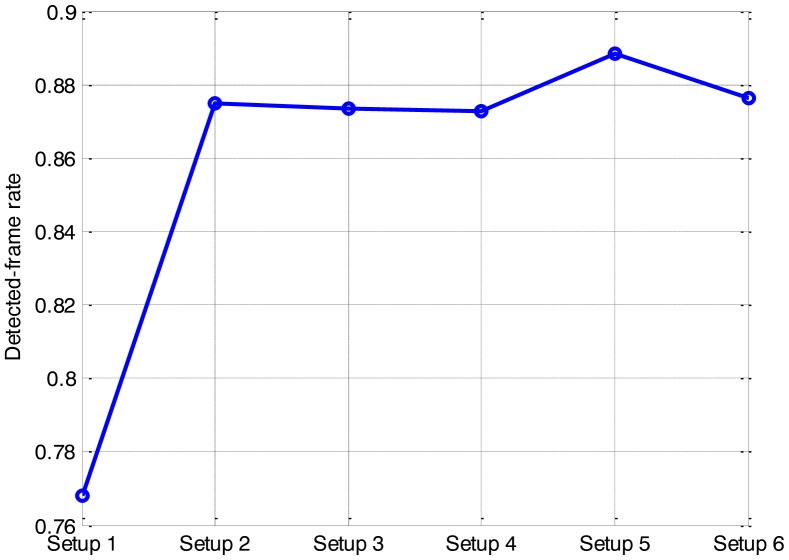
Detection sensitivity of the different setups.

**Figure 7.**
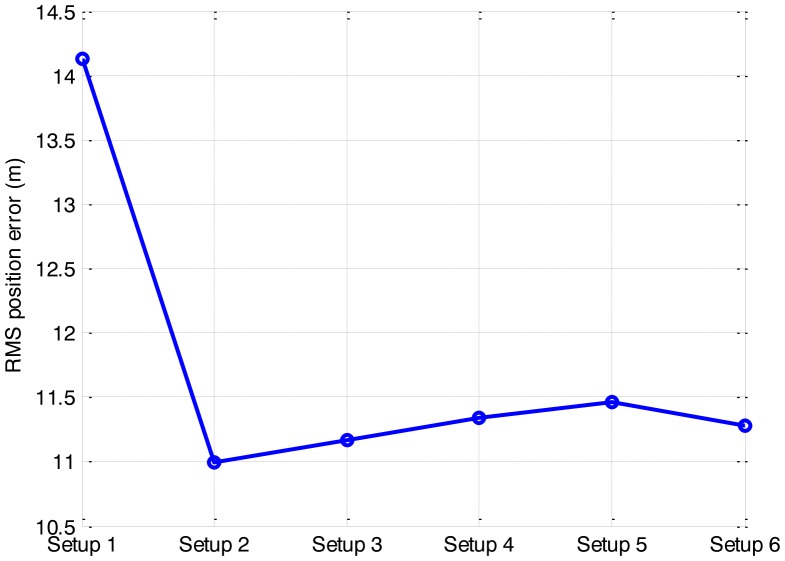
Estimation accuracy of the different setups.

Six setups are used to represent six different sizes of the sub-area. Corresponding *P_sub_* and computation requirements are compared in Table 2. The detected-frame rate and the RMS position error of the different setups are given in Figures 6 and 7, respectively.

As anticipated, the substitution of the sub-area for the whole area to calculate the likelihood ratio improves the computational efficiency significantly. As seen from Table 2, the smaller the size of the sub-area is, the less CPU time is required. However, the effect of the sub-area on the detection and tracking performance is complicated. It shows an obvious performance degradation in setup 1, and negligible performance changes among other setups. It is demonstrated that substituting a single cell for the whole area to calculate the likelihood ratio improves the efficiency most, but also degrades the accuracy severely. Compared other setups, setup 2 is optimal to substitute for the whole area, as it consumes comparable CPU time with setup 1 but with little loss in accuracy.

Therefore, compromising between efficiency and accuracy, the size of the sub area 3 × 3 bins, taking up more than 80% power of the measurement function, is the optimal for the efficient application of the PF in this paper. It would result in a noticeable improvement on the efficiency, with little loss in the accuracy.

**Figure 8.**
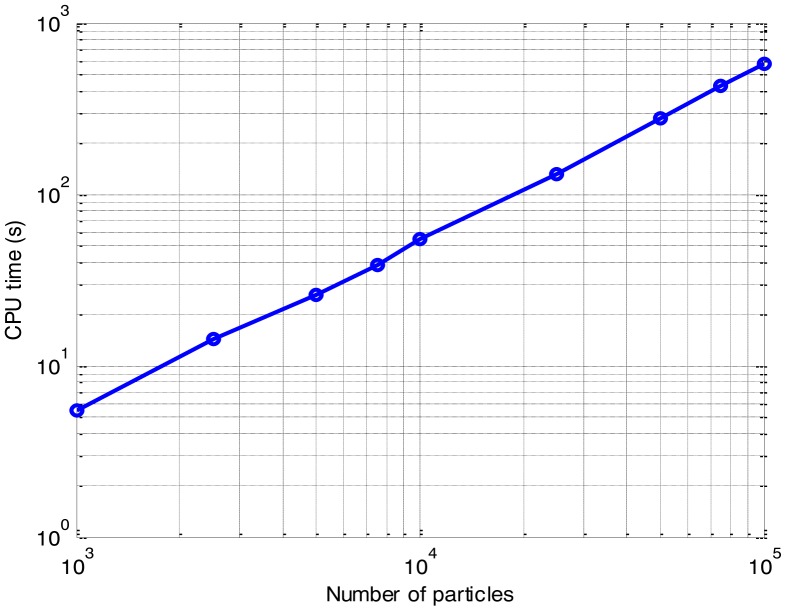
Computation requirements of the different number of particles.

**Figure 9.**
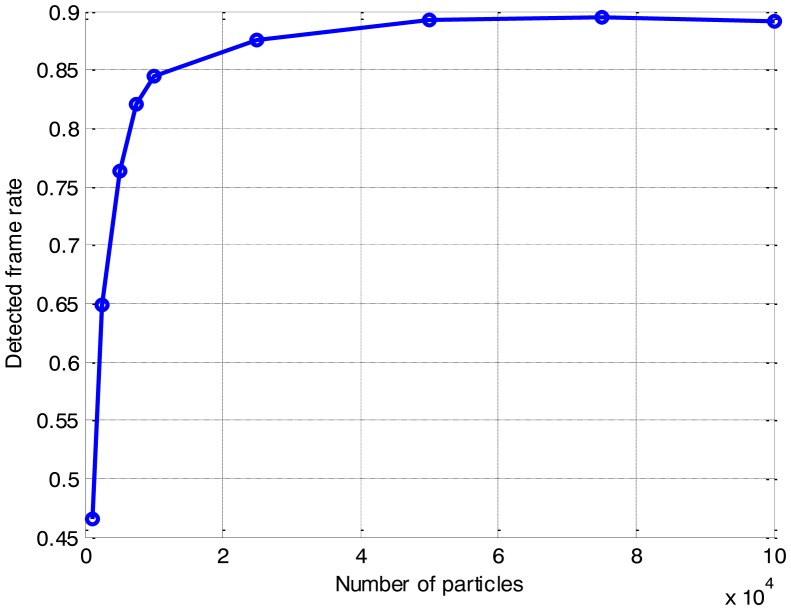
Detection sensitivity of the different number of particles.

**Figure 10.**
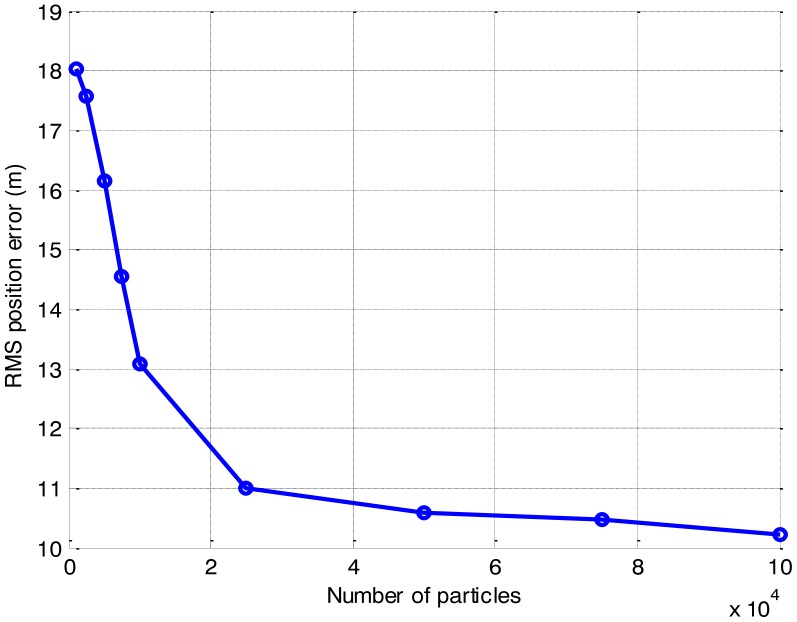
Estimation accuracy of the different number of particles.

The effect of the number of particles on the CPU time is presented in Figure 8. The detected-frame rate and the RMS position error of the different number of particles are also compared respectively, in Figures 9 and 10.

As anticipated, the CPU time increases with the growth of the number of particles. It is shown that the CPU time grows linearly with the increase of the number of particles, while the detection and tracking performance are also improved as the number of particles increases. As shown in Figures 9 and 10, when the number of particles is more than 25,000, both the detected-frame rate and the RMS position error fall to a steady level with little improvement, but the CPU time is growing continually. It is demonstrated that a pertinent choice of the number of particles is significant, since the improvement on the accuracy with the increase of the number of particles is finite.

Therefore, compromising between efficiency and accuracy, for multi-frame SAR images with 200 × 32 bins, a number of particles of 25,000 is optimal for the PF implementation in this paper. This would result in a noticeable improvement in the efficiency, with little loss in the accuracy.

## 6. Conclusions

An approach to detection and tracking of moving targets using SAR images, is proposed and evaluated in this paper, based on the PF-based TBD algorithm. The signal model of the SAR moving target is incorporated into the PF-based TBD algorithm for the first time, which is capable of resolving the ambiguity in target azimuth position and radial velocity while tracking. The proposed approach is capable to detect a moving target with a SNR as low as 7 dB with a detected-frame rate of 0.7 for the false-report rate below 0.005, and track with a RMS position error smaller than 16 m. The proposed TBD algorithm outperforms the traditional TAD algorithm when the SNR is below 14 dB. An efficient calculation method of the likelihood ratio is proposed to improve the efficiency of the PF. Due to the inherent characteristics of the SAR measurement function, a small sub-area has taken up the majority of the power. The substitution of the sub-area for the whole area to calculate the likelihood ratio is demonstrated to improve the efficiency significantly, with little loss in the detection and tracking performance. Moreover, a pertinent choice of the number of particles is crucial for the efficient PF application. It is demonstrated that the computation requirements increase linearly with the growth of the number of particles, while the detection and tracking performance fall to a steady level. The optimal number of particles for this simulation is achieved via Monte Carlo trials, providing a reference for other similar investigations.

## Figures and Tables

**Table 1.** False-report rates and computation requirements of the different algorithms.

	**TBD Algorithm**	**TAD Algorithm**
**False-report Rate**	0.005	0.018
**CPU Time**	131.0 s	9.2 s

**Table 2.** Normalized powers and computation requirements of the different setups.

	*Setup 1*	*Setup 2*	*Setup 3*	*Setup 4*	*Setup 5*	*Setup 6*
*N_wa_* × *N_wr_*	1 × 1	3 × 3	5 × 5	9 × 9	32 × 32	*N_a_* × *N_r_*
*P_sub_*	42%	83%	91%	95%	99%	1
*CPU Time*	119.2 s	128.1 s	132.3 s	144.2 s	317.5 s	1508.0 s
